# Robotic-assisted reversal of Hartmann’s procedure for diverticulitis

**DOI:** 10.1007/s11701-014-0458-z

**Published:** 2014-03-16

**Authors:** Nicola de’Angelis, Emanuele Felli, Daniel Azoulay, Francesco Brunetti

**Affiliations:** Digestive Surgery and Liver Transplant Unit, Henri-Mondor Hospital, Université Paris Est, UPEC, 51 avenue du Maréchal de Lattre de Tassigny, 94010 Créteil, France

**Keywords:** Robotic surgery, Hartmann’s procedure, Hartmann’s reversal, Diverticulitis

## Abstract

The minimally invasive laparoscopic approach for the reversal of Hartmann’s procedure (HP) has been shown to be a safe and feasible approach associated with low morbidity and fast recovery. Robotic surgery has not yet been described for HP reversal. We report the case of an 84-year-old man originally operated on in an emergency setting by conventional HP for complicated diverticulitis who underwent a robotic-assisted HP reversal. The surgical procedure and the post-operative follow-up were uneventful, with low post-operative pain, early return to bowel function, and discharge at day 3. The robotic surgery appeared to be a safe, feasible, and valuable approach for HP reversal.

## Introduction

The Hartmann’s procedure (HP) was first described in 1921 [1] and consisted of a sigmoidectomy with a rectal stump closure and a terminal colostomy. Initially, it was performed in left-sided colonic carcinoma cases, but current indications include complicated diverticulitis, traumatic lesions, and perforated recto-sigmoid and volvulus [2]. The reestablishment of intestinal continuity after HP (i.e., Hartmann’s Reversal, HR) is a major surgical procedure that can be performed in only one-third of cases [3, 4]. HR is still associated with a serious risk of surgical morbidity (in up to 50 % of cases), including a high rate of anastomotic leakage, and a considerable mortality risk (range 4–10 %) [4, 5, 6, 7], whether performed by conventional or laparoscopic approaches. However, since the first use of laparoscopy for HR in 1993 [8], several studies have demonstrated that laparoscopy compares favorably with the conventional open procedure in terms of earlier bowel function restoration, less post-operative pain, a more rapid return to a normal diet, and reduced morbidity [7, 9]. These advantages are more likely related to the minimally invasive technique. Robotic surgery, which to our knowledge has not yet been described for HR, could be a feasible and valuable approach. We report a robotic-assisted HP reversal in a patient previously operated on for a complicated diverticulitis.

## Case report

An 84-year-old man with a history of coronary artery disease, type I diabetes, chronic kidney disease, and a poor nutritional status, was operated on for perforated diverticulitis (Hinchey IV) in May 2013. At that time, the patient arrived hemodynamically unstable and was infused with continuous norepinephrine (0.1 μg/kg/min). HP via laparotomy was then performed on the patient in an emergency setting. The post-operative period was uneventful, and the patient was discharged at day 9. Five months later, once the patient achieved an optimal performance status, a robotic HR via da Vinci^®^ Si Surgical System was planned. Pre-operatively, the patient underwent an anatomical evaluation by computed tomography and colonoscopy of the remaining proximal colon and rectal stump.

### Operative technique

The patient underwent a bowel preparation (including enemata to empty the rectal stump) 24 h before surgery and received perioperative broad-spectrum parenteral antibiotics and subcutaneous low-molecular-weight heparin. No ureteric catheter was used. The surgical protocol was similar to that applied for the laparoscopic HR [9].

**Fig. 1:**
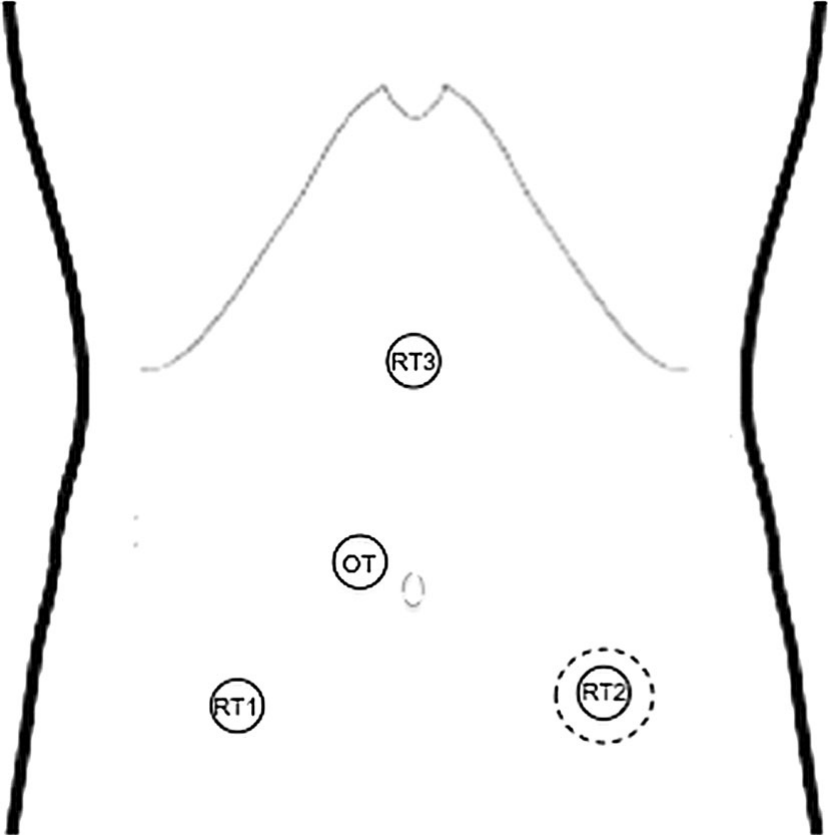
Schematic of the trocar placement. Robotic trocars 1 (RT1), 2 (RT2), and 3 (RT3) were 8-mm trocars. The optical trocar (OT) was a 12-mm trocar. The dotted line at the RT2 level represents the Alexis Laparoscopic System^®^ placed at the site of the previous colostomy

**Fig. 2:**
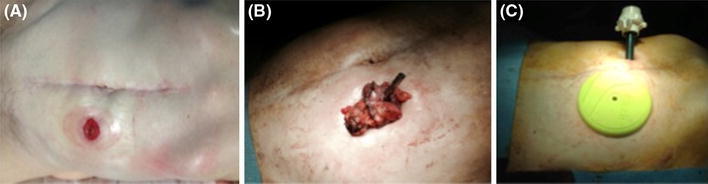
Intra-operative photographs. **a** The colostomy. **b** After mobilization and colostomy excision, the stapler anvil is introduced into the proximal colon by purse string suturing. **c** The Alexis Laparoscopic System^®^ and optical trocar placement

The patient was placed in a modified lithotomy position, with a 30° Trendelenburg, and tilted to the right side. The first surgical step was the excision of the colostomy and bowel mobilization out of the abdomen. Then, the stapler anvil was introduced into the proximal colon by purse string suturing, as described previously [10]. The bowel was returned to the abdominal cavity after all existing adhesions were dissected. By using a small Alexis Laparoscopic System^®^ (Applied Medical, CA, USA), the abdominal wound of the previous colostomy site was used for pneumoperitoneum establishment and for the set-up of one temporary optical trocar, which allowed for the placement of the robotic arms and camera under direct vision. The first 8-mm robotic trocar was placed at the intersection point between the right midclavicular line and the line between the umbilicus and the right superior iliac spine (RT1). Then, a 12-mm optical trocar for the camera was inserted 3 cm right of and lateral to the umbilicus (OT). At this point, the other robotic arms were placed under direct vision by the OT camera, and the previously used optical trocar in the colostomy site was replaced by the second 8-mm robotic trocar (RT2). The third robotic trocar was inserted 5 cm below the xiphoid process on the right side of the falciform ligament (RT3). The da Vinci^®^ robot was docked into the ports on the left side of the patient with an angle of 30°–40° to the operating bed (Figs. 1, 2).

The dissection began with the adhesiolysis. When the target of the operation was in the upper quadrants, fenestrated bipolar forceps were used on the RT3; a hook with monopolar cautery was used on the RT1, and a grasping retractor was used on the RT2. When the target of operation was in the pelvis, fenestrated bipolar forceps were used on the RT2, and a grasping retractor was used on the RT3.

The colonic left flexure was mobilized to ensure a tension-free anastomosis without dissection of the inferior mesenteric vein origin. Then, the rectal stump was dissected to achieve an end-to-end anastomosis and to avoid bladder injury. Finally, a colorectal anastomosis was performed mechanically (29 mm) without a stoma diversion. The peri-operative anastomosis was controlled by using an air-leak test. The colostomy wall defect was closed using three layers of interrupted non-absorbable sutures. No abdominal drain was used. The operative time was 190 min, and the estimated blood loss was 210 mL.

The post-operative period was uneventful; flatus was observed at day 1, and a normal diet was restored at day 2. The patient was discharged at post-operative day 3. At the 2-month follow-up, the patient was in good health.

## Discussion

More frequently, in the last decade, HR has been performed by a mini-invasive technique, which has become the preferable approach in many cases. Robotic surgery has not yet been applied for HR; however, it can be expected to provide advantages similar to laparoscopy, such as reduced surgical trauma and early recovery.

The presently reported robotic surgery was the first HR intervention, and the fourth colorectal procedure performed in our unit since the robotic surgery program began in September 2013. Although described as a time-consuming and challenging technique, especially when learning the technique [11, 12], the robotic HR had an operative time that did not exceed the mean duration of previously reported laparoscopic HR in our unit [9] and was not longer than ranges reported in the literature [7]. The described robotic HR followed the protocol that is routinely applied for laparoscopic HR, in which the colostomy site is used for the set-up of the first optical trocar. This technique can help the set-up of remaining trocars, not only allowing for direct vision but also providing, if necessary, a laparoscopic-assisted adhesiolysis to achieve the correct trocar placement before docking of the robotic arms. In our patient, this was not necessary, and robotic adhesiolysis and the rectal stump dissection were easy, uneventful, and smoother than by laparoscopy. This is related to the fact that the da Vinci^®^ Si Surgical System scores over conventional laparoscopy by providing 3D vision and instruments with EndoWrist technology, which enables the surgeon to perform very precise dissections. These technical advantages may also contribute to reducing the conversion rate from a minimally invasive approach to laparotomy [7].

This robotic surgery was associated with a good and uneventful post-operative follow-up; the patient reported mild post-operative pain, which was managed without morphine, and showed a fast post-operative recovery. He was discharged at day 3. In our experience, these clinical outcomes are comparable to those associated with the laparoscopic approach [9, 13].

In the learning curve process, HP reversal may represent a valuable training intervention in robotic colorectal surgery.

## Conclusion

The robotic surgery appears to be a safe, feasible, and valuable approach for HP reversal.
